# Smart campus: Data on energy generation costs from distributed generation systems of electrical energy in a Nigerian University

**DOI:** 10.1016/j.dib.2018.02.022

**Published:** 2018-02-16

**Authors:** Joshua O. Okeniyi, Aderemi A. Atayero, Segun I. Popoola, Elizabeth T. Okeniyi, Gbenga M. Alalade

**Affiliations:** a Mechanical Engineering Department Covenant University, Ota, Nigeria; b Chemical, Metallurgical and Materials Engineering Department, Tshwane University of Technology, Pretoria, South Africa; c Department of Electrical and Information Engineering, Covenant University, Ota, Nigeria; d Petroleum Engineering Department, Covenant University, Ota, Nigeria; e Department of Architecture, Covenant University, Ota, Nigeria; f Physical Planning and Development Unit, Covenant University, Ota, Nigeria

**Keywords:** Smart campus, Energy consumption, Energy efficiency, Load forecasting, Energy management, Learning analytics, Nigerian university, Education data mining

## Abstract

This data article presents comparisons of energy generation costs from gas-fired turbine and diesel-powered systems of distributed generation type of electrical energy in Covenant University, Ota, Nigeria, a smart university campus driven by Information and Communication Technologies (ICT). Cumulative monthly data of the energy generation costs, for consumption in the institution, from the two modes electric power, which was produced at locations closed to the community consuming the energy, were recorded for the period spanning January to December 2017. By these, energy generation costs from the turbine system proceed from the gas-firing whereas the generation cost data from the diesel-powered generator also include data on maintenance cost for this mode of electrical power generation. These energy generation cost data that were presented in tables and graphs employ descriptive probability distribution and goodness-of-fit tests of statistical significance as the methods for the data detailing and comparisons. Information details from this data of energy generation costs are useful for furthering research developments and aiding energy stakeholders and decision-makers in the formulation of policies on energy generation modes, economic valuation in terms of costing and management for attaining energy-efficient/smart educational environment.

**Specifications Table**

**Table:** 

Subject area	*Engineering*
More specific subject area	*Electrical Engineering, Mechanical Engineering, Engineering Economics, Engineering Physics*
Type of data	*Tables, graphs, figures and spreadsheet files*
How data was acquired	*Monitoring, logging in records and cumulated for each month of the year*
Data format	*Raw, analyzed*
Experimental factors	*Data monitoring and logging were performed manually rather than being automated*
Experimental features	*Ordered statistics was employed in combination with cumulative distribution fitting analyses, Kolmogorov-Smirnov goodness-of-fit statistics (K-S GoF) was employed for test-of-significance of the data distribution fitting*
Data source location	*The dataset of energy generation cost provided in this article were collected at Covenant University, Canaanland, Ota, Nigeria (Latitude6.6718°N, Longitude3.1581°E)*
Data accessibility	*A comprehensive dataset of energy generation cost is provided in this article.*

**Value of the data**

• Accessibility to datasets of energy generation cost of a distributed generation system of electrical energy production using gas-fired and diesel engine generators that could be used for fostering systems of practical data-driven research in the understanding of energy cost modeling valuations and how this can be improved towards efficient integration of energy generation for a smart university campus [1], [2], [3], [4], [5].
• Costs of energy generation data that could be employed for energy generation planning in the development of new energy generation plants as well as in the decision making of how to combine energy generation systems for electricity consumers in a smart university campus [6], [7].
• Availability of energy generation costs that could be used for estimations of energy generation cost parameters and cost concepts, such as *levelised* cost of electric energy, society cost of electric energy, returns on investment on energy generating plants, projection of future energy costs for budget purposes, for energy stakeholders and decision-makers of energy production in a smart university campus [8], [9], [10].
• Applicability and/or developmental prospects of Smart Electrical Energy Network (SEEN) for a stronger, more sustainable controls of centralized distributed generation of electric energy system via systems of the electric energy generation costing for a smart university campus [3], [4], [11].

## 1 Data

Although rising energy demand remains a global issue, it is still a well-known fact that a large and increasing portion of the populace in a developing country like Nigeria has no access to the national grid generated electric power [12], [13], [14], [15], [16].Therefore, for many establishments and households in the country, resorting to off-grid self-generation of electric energy remains the only option for maintaining comfortable working and living environments. In a smart university campus, for instance, adequate electricity generation is required for ensuring comfortable indoor conditions for staffs and students in the office spaces, classrooms, as well as in the staffs and students accommodations [17]. Apart from these, sustainable educational processes necessitate that the electric load requirements of facilities, equipment and support services of the smart university community be satisfied to acceptable standards [5]. In meeting such a demand, the self-generation of electricity will attract an additional cost of educational service rendering which definitely requires innovative approach for keeping such cost of electric energy generation from being exorbitant or unaffordable. Therefore, data that is being made available to the public in the present paper is constituted of pertinent information on the electric energy generation cost from distributed energy requirement in Covenant University, Ota, Nigeria; an ICT-driven smart university campus. The data were monitored and compiled for 12 consecutive months spanning January-December, 2017, from gas-fired turbine system and diesel-fueled electric-generating units, used for backup applications that ensure continuous electric energy generation for meeting the energy needs of the university campus [3], [18], [19].

Table 1 contains data of electric energy generated for consumers in the university campus and the cost of generating the electric energy using the gas-fired turbine tower and the diesel-fuel generators on campus. Also included in the table are the running hours of employing the diesel fuel unit as backups, the volume, in liters (L), of the diesel fuel consumed for every month in the year 2017, as well as the summation of all the data presented in the table for the year.

**Table 1:** Electric energy generation data at Covenant University for the Year 2017.

**S/N**	**Month**	**Days Monitored (day)**	**Time (h)**	**Electric energy (kW h)**	**Maintenance Cost (€)**	**Gas-fired Turbine**	**Diesel-fired Generator**		
						**Cost (€)**	**Diesel Cost (€)**	**Diesel (L)**	**Running time (h)**
1	Jan	31	744	906,296.00	0.00	64,953.98	30,241.78	84,754.80	96.52
2	Feb	28	672	726,964.74	0.00	57,159.76	74,213.23	207,988.00	234.35
3	Mar	31	744	1,225,867.80	0.00	131,607.46	39,406.64	110,440.00	125.83
4	Apr	30	720	1,374,884.00	0.00	162,524.20	9140.46	25,616.80	26.27
5	May	31	744	1,130,266.50	0.00	127,546.57	8791.92	24,640.00	26.05
6	Jun	30	720	652,749.00	6553.62	71,632.81	4433.64	12,425.60	14.20
7	Jul	31	744	675,810.20	0.00	75,077.52	3202.77	8976.00	10.33
8	Aug	31	744	565,981.50	1930.99	73,950.10	19,467.82	54,560.00	62.63
9	Sep	30	720	974,627.00	0.00	112,526.22	13,187.88	36,960.00	42.05
10	Oct	31	744	828,387.17	1090.90	87,229.96	3921.40	10,990.00	14.15
11	Nov	30	720	712,010.00	743.18	48,545.70	12,310.12	34,500.00	50.00
12	Dec	22^a^	528	409,340.00	79,721.60	27,909.29	16,815.62	47,127.00	68.05

a 22 days were monitored in December 2017, after which the university proceeded to end-of-year break.

**Fig. 1:**
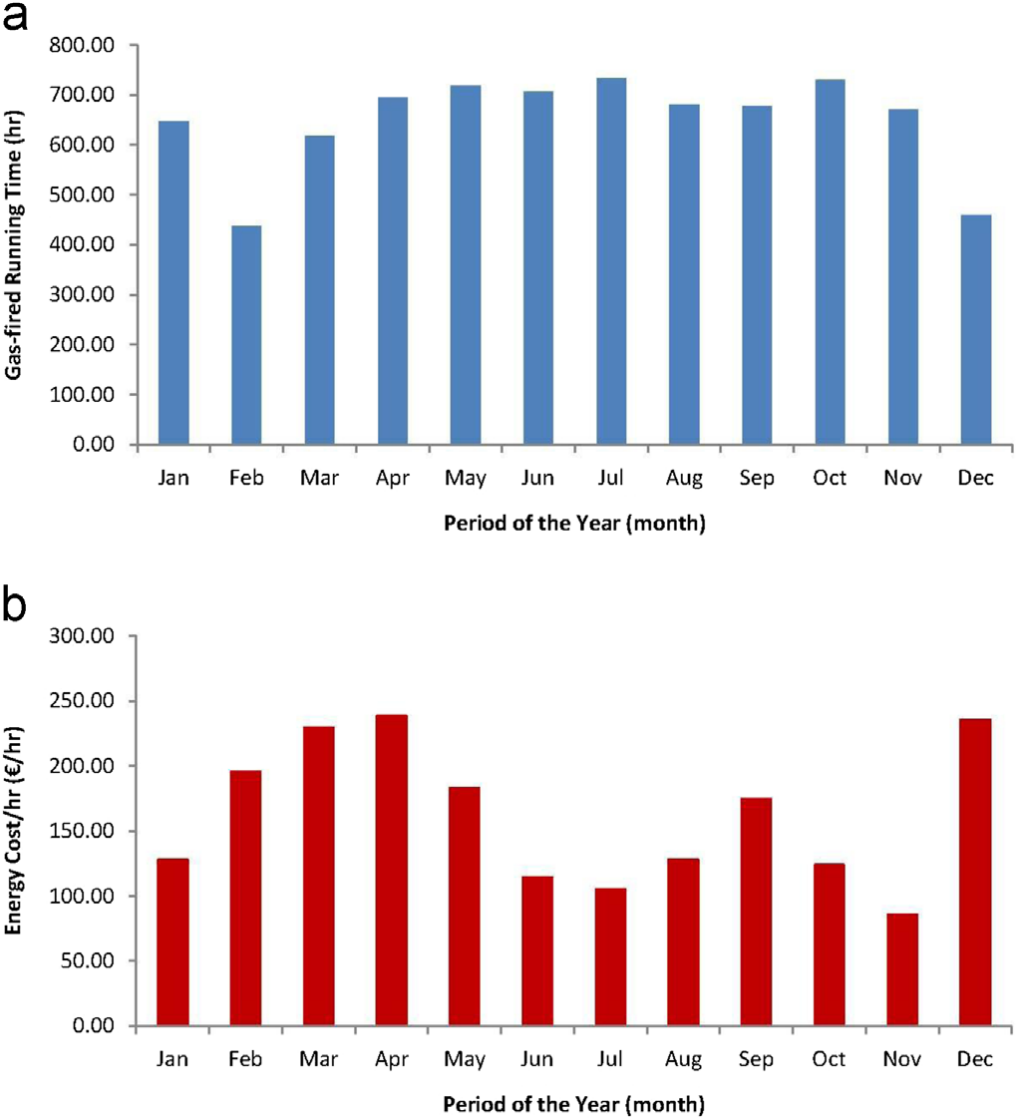
Plots for the monthly period of the year 2017 of estimated data in Covenant University, Ota, Nigeria, of (a) running time of gas-fired turbine generator, and (b) electric energy generation cost per hr.

Directly proceeding from Table 1 includes further data of running time (h) of the gas-fired turbine and the electric energy generation cost per hour (€/h) for the period spanning January-December 2017 was estimated and these parameters are respectively presented in Fig. 1(a) and (b).

## 2 Experimental design, materials and methods

**Fig. 2:**
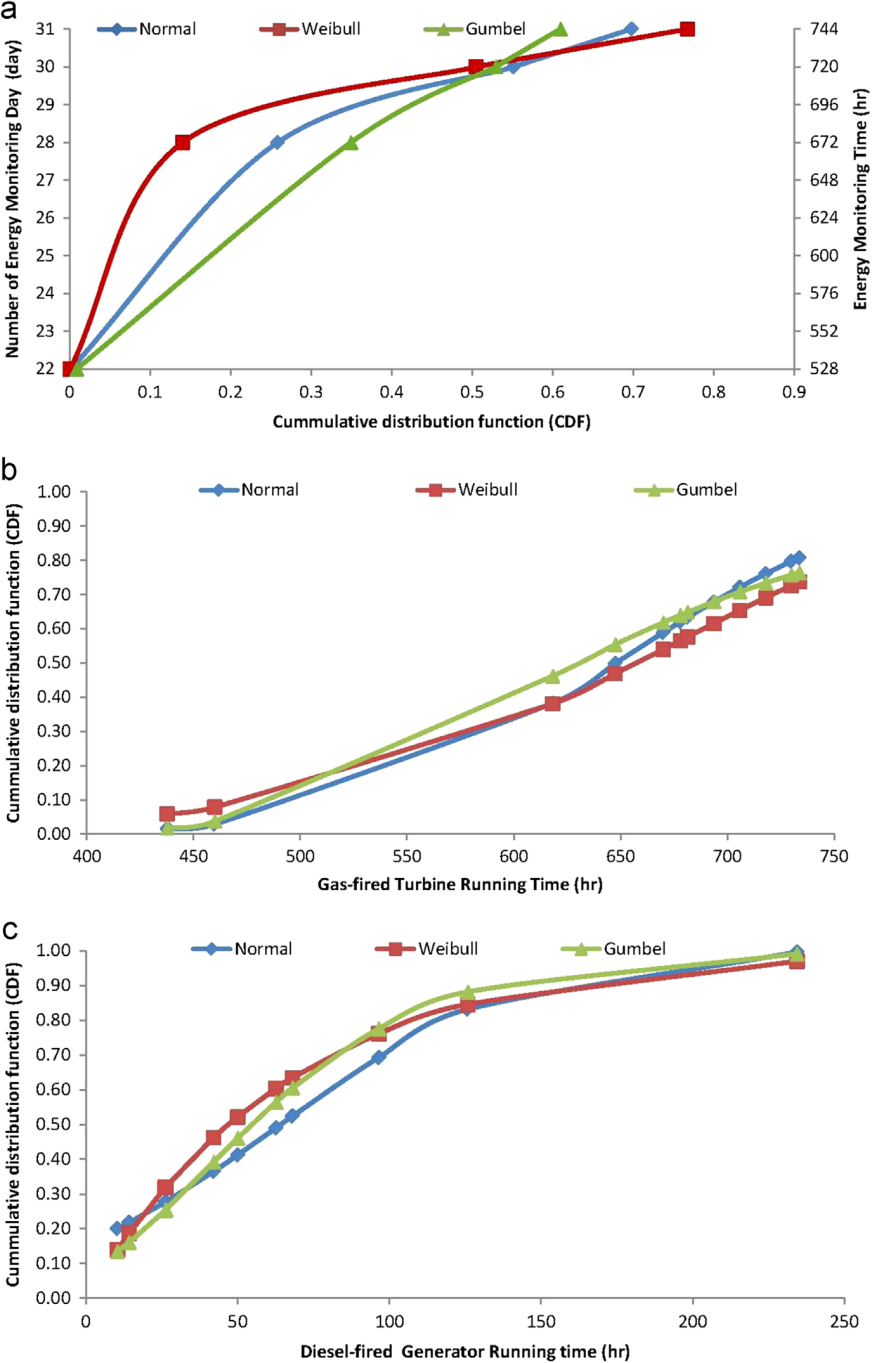
Analyzed cumulative distribution functions of timeframes for electricity generation cost data.

**Fig. 3:**
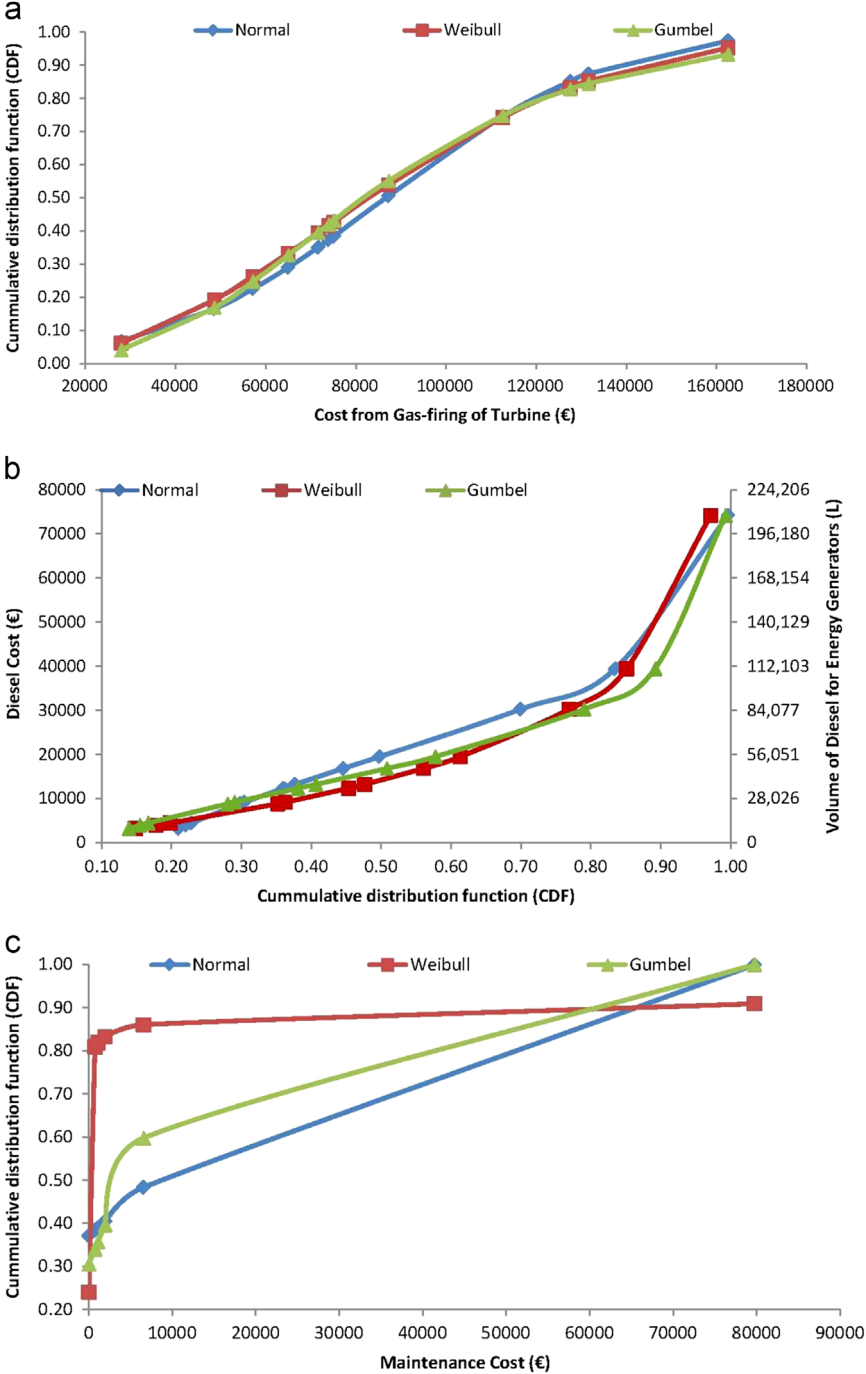
Analyzed cumulative distribution functions of operations and maintenance requirements for electricity generation cost data.

**Fig. 4:**
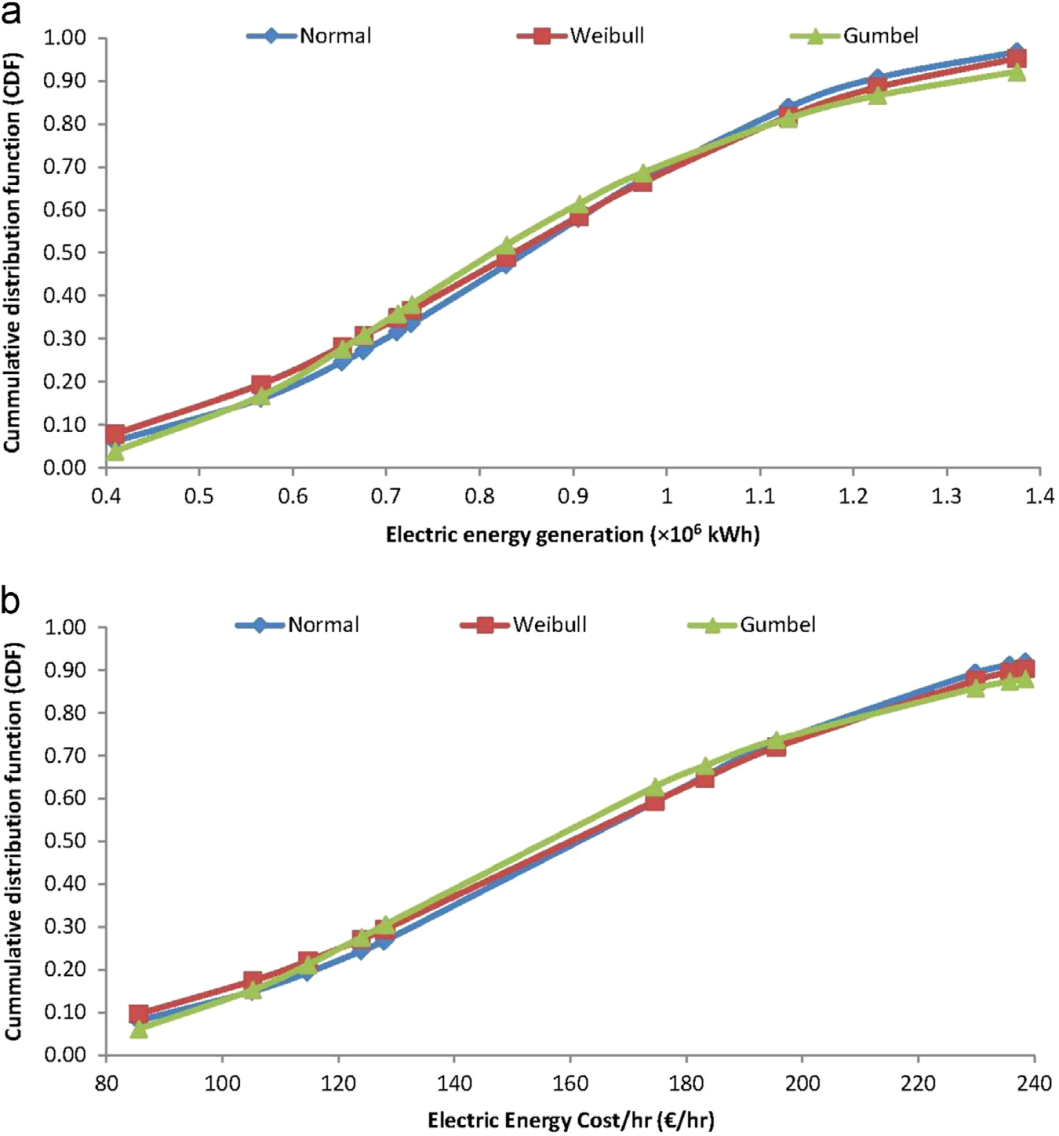
Analyzed cumulative distribution functions of energy effects from the generation cost data.

As detailed, the electric energy generation cost data were monitored and cumulated for each month of the year 2017, at Covenant University, Ota, Nigeria, as a part of continuous data sourcing and management in the smart university campus. The generation of the distributed electric energy is required for the maintenance of conducive/comfortable education and working environments that promote the well-being of students and staffs in the fully residential university [5], [17]. For aiding understanding and reusability of the electricity generation cost data, analyses of each variable for the cost data, presented in Table 1 and in Fig. 1 are conducted using applications of ordered statistics and cumulative distribution function models of the Normal, Weibull and Gumbel distributions. For these cumulative distribution function analyses, which require estimations, especially, of the shape and scale parameters of the Weibull distribution and the location and scale parameters of the Gumbel distribution models [12], [20], [21], maximum likelihood estimation, as detailed in [22], [23], [24], [25], [26], were employed. These analyses gave the data of cumulative distribution fittings of the Normal, Weibull and Gumbel probability distributions which are presented in Fig. 2, for timeframes, Fig. 3 for operation and maintenance and Fig. 4 for effects and materials ensuing from the electric energy generation cost data.

The cumulative distribution function analyses of the energy generation cost data necessarily lead to the estimations of the descriptive statistics parameters to which was combined the testing of significance of the compatibility of the electric energy generation cost data to each of the statistical distributions [27], [28], [29], [30], [31]. For the distribution fitting compatibility testing, the Kolmogorov-Smirnov goodness-of-fit statistics [31], [32], [33], [34], [35] was used. Data from the descriptive statistics and the K-S *p*-value, i.e. the probability value obtained from the Kolmogorov-Smirnov goodness-of-fit statistical analyses, are presented in Table 2 for the Normal, Table 3 for the Weibull and Table 4 for the Gumbel distributions.

**Table 2:** Descriptive statistics of electrical energy generation cost data using the Normal distribution.

**Energy generation cost parameter**	**Normal Mean (*****μ***_***N***_**)**	**Normal Standard Deviation (*σ****_**N**_***)**	**Probability(*****µ***_***N***_**)**	**K-S *p*-value**
Days Monitored (day)	29.67	2.57	0.5	0.0414
Time (h)	712.00	61.69	0.5	0.0414
Electric energy (kW h)	848,598.66	284,884.43	0.5	0.9362
Maintenance cost (€)	7,503.36	22,820.15	0.5	0.0145
Cost (€)	86,721.96	39,177.89	0.5	0.6755
Gas-fired turbine running time (h)	647.80	98.79	0.5	0.3535
Diesel cost (€)	19,594.44	20,362.86	0.5	0.3680
Diesel (L)	54,914.85	57,068.40	0.5	0.3680
Diesel-fired generator running time (h)	64.20	64.18	0.5	0.5081
Energy cost/hr (€/h)	161.91	54.49	0.5	0.4730

**Table 3:** Descriptive statistics of electrical energy generation cost data using the Weibull distribution.

**Energy generation cost parameter**	**Weibull Mean (*****μ***_***W***_**)**	**Weibull Standard Deviation (*σ****_**W**_***)**	**Probability(*****µ***_***W***_**)**	**K-S *p*-value**
Days Monitored (day)	29.75	1.66	0.4413	0.1000
Time (h)	713.93	39.88	0.4413	0.1000
Electric energy (kW h)	844,736.28	308,508.43	0.5098	0.9616
Maintenance cost (€)	3,107,591,209.09	7,883,020,667,225.09	0.9960	0.0913
Cost (€)	86,813.00	41,644.57	0.5349	0.8857
Gas-fired turbine running time (hr)	648.82	82.44	0.4567	0.4919
Diesel cost (€)	20,635.11	20,925.01	0.6343	0.9207
Diesel (L)	57,831.41	58,643.89	0.6343	0.9207
Diesel-fired generator running time (h)	67.44	66.94	0.6310	0.9472
Energy cost/hr (€/h)	161.50	57.61	0.5079	0.6405

**Table 4:** Descriptive statistics of electrical energy generation cost data using the Gumbel distribution.

**Energy generation cost parameter**	**Gumbel Mean (*****μ***_***G***_**)**	**Gumbel Standard Deviation (*σ****_**G**_***)**	**Probability(*****µ***_***G***_**)**	**K-S *p*-value**
Days Monitored (day)	30.50	5.10	0.5704	0.0371
Time (h)	731.91	122.47	0.5704	0.0371
Electric energy (kW h)	869,413.46	335,572.30	0.5704	0.9857
Maintenance cost (€)	5,870.67	10,077.30	0.5704	0.0131
Cost (€)	89,316.12	45203.28	0.5704	0.9055
Gas-fired turbine running time (h)	668.46	163.16	0.5704	0.2329
Diesel cost (€)	19,196.39	16,305.12	0.5704	0.8451
Diesel (L)	53,799.29	45,696.30	0.5704	0.8451
Diesel-fired generator running time (h)	63.43	53.52	0.5704	0.9622
Energy cost/hr (€/h)	165.23	63.69	0.5704	0.7182
